# Occupational Stress and Its Economic Cost in Hong Kong: The Role of Positive Emotions

**DOI:** 10.3390/ijerph17228601

**Published:** 2020-11-19

**Authors:** Oi Ling Siu, Cary L. Cooper, Lara C. Roll, Carol Lo

**Affiliations:** 1 Department of Applied Psychology, Lingnan University, Hong Kong, China; lara.roll@kuleuven.be; 2 Alliance Manchester Business School, University of Manchester, Manchester M13 9PL, UK; cary.cooper@manchester.ac.uk; 3 Department of Work, Organisational and Personnel Psychology, KU Leuven, 3000 Leuven, Belgium; 4 Optentia Research Focus Area, North-West University (VTC), Vanderbijlpark 1174, South Africa; 5 Occupational Safety and Health Council, Hong Kong, China; carollo@oshc.org.hk

**Keywords:** absenteeism, economic cost, occupational stress, presenteeism, positive emotions

## Abstract

There has been less research on the costs of occupational stress attributed to certain job stressors in Chinese contexts. This study identified and validated common job stressors and estimated the economic cost in Hong Kong. The role of positive emotions in alleviating the economic costs of job stressors was also examined. Both qualitative and quantitative approaches were adopted. The findings obtained from five focus group discussions and a survey validated five common job stressors: Job insecurity; quantitative workload; organizational constraints; interpersonal conflicts; and work/home interface. A total of 2511 employees were surveyed, with 2032 valid questionnaires returned (925 males, 1104 females, and 3 unidentified, whose ages ranged from 18 to 70 years). The economic costs were estimated by combining the costs of absenteeism, presenteeism, and medical expenses. Absenteeism mainly caused by job stressors of the work/home interface, job insecurity, and quantitative workload accounted for an annual economic cost of HK$550 million to HK$860 million. The annual economic cost due to presenteeism mainly caused by job stressors of job insecurity, interpersonal conflict, quantitative workload, and organizational constraints ranged from HK$1.373 billion to HK$2.146 billion. The cost of medical treatments associated with occupational stress was HK$2.889 billion to HK$4.083 billion. Therefore, the total annual economic cost of occupational stress was approximately HK$4.81 billion to HK$7.09 billion. Positive emotions, representing a less explored individual factor in the cost of occupational stress studies, was found to be negatively correlated with presenteeism and buffered the negative impact of job stressors on absenteeism. The theoretical contributions and practical implications of findings are discussed.

## 1. Introduction

Occupational stress is costly. The influence of occupation-related stress on workplace productivity and the extended economy has become critical. The economic implications mainly include stress-related absenteeism (i.e., failure to attend work), stress-related presenteeism (i.e., attending work, but performing at a suboptimal capacity), and annual medical expenses (induced medical costs), among others [1,2,3,4,5]. It is imperative to minimize these stress-related negative consequences in the workplace. However, there has been more research on absenteeism than presenteeism, and few studies have examined the economic costs of both absenteeism and presenteeism empirically and simultaneously to generate a more holistic picture. 

There have been studies illustrating how individual factors such as personality and attitude towards work [6,7] affect attendance behavior. According to the broaden-and-build theory [8], positive emotions can form an upward spiral by inspiring thoughts and behaviors and accruing their personal resources, such as psychological resources. In turn, according to job demands–resources theory [9], those personal resources can directly lead to better job performances through the motivation process and indirectly alleviate the negative impacts of job demands through the strain process. Therefore, positive emotions may directly or indirectly decrease the economic costs of job stress. To date, studies that have examined the role of positive emotions in alleviating the economic cost of occupational stress are scarce.

Research on the costs of stress attributed to certain job stressors has mainly been conducted in Western contexts, such as the USA [10], Australia [11], Spain [12], and in a review paper developed in the UK [1]. The economic costs of occupational stress in Chinese or Asian contexts remain unclear. Hong Kong is an area with a mix of both Western and Eastern cultures, though the majority of residents are Chinese. Exploring the costs of stress in Hong Kong will add to the literature to provide convergent validity and generalizability for the Western findings. Most importantly, this research will serve as the basis for similar research in other Eastern contexts, in order to arouse public awareness across Asia and enhance the competitiveness of Asian societies by reducing the cost of occupational stress.

To address these research gaps, in this study, we will identify and validate the major job stressors and estimate the economic cost of occupational stress in Hong Kong, as well as explore the role that positive emotions play in alleviating the economic costs of job stressors. The current study will advance the research methodology used in estimating the cost of occupational stress by adopting both qualitative and quantitative approaches.

## 2. Theoretical Background

### 2.1. Global Economic Impact of Occupational Stress

Occupational stress causes economic costs due to health impairment at both individual and organizational levels [2,12,13]. At the individual level, occupational stressors are associated with increased economic costs due to productivity loss and medical-related costs, as well as a decreased salary [12,14]. Work-related stress has been consistently found to cause anxiety or depression disorders among employees [15], which in turn results in productivity impairment and costs for seeking medical help [14]. Employees with work-related impairments, such as insomnia [16] and musculoskeletal disorders [17], also receive lower salaries.

At the organizational level, occupational stress can lead to costs of productivity loss due to an employee’s absenteeism or presenteeism and the costs of medical insurance claims. When employees are sick, organizations are required to pay part of the medical fees. In 2011 and 2012, occupational stress and related psychological symptoms caused a total of 10.4 million lost working days, with an average of 24 absent days per employee, in the UK [18]. Presenteeism is another source of productivity loss [19], constituting a great proportion of economic loss, together with absenteeism [1]. 

### 2.2. The Cost of Absenteeism

Absenteeism is related to work stress, such as workload, job control, conflict of roles, leadership quality, shift work, and limited career progression [20,21,22,23,24,25]. In the literature, the cost of productivity loss (economic impact of occupational stress) due to absenteeism has typically been calculated in two ways: The lost wages method and the friction cost method [26]. The lost wages method, also called the human capital method, involves multiplying the number of days lost by the absent employee’s daily salary or by the organization’s average wages, assuming that the wages of absent employees represent the cost of organizational productivity [27]. This approach provides a rough estimate [28] because it overlooks the possibility that other employees could compensate to restore the normal productivity. The frictional cost approach attempts to quantify the effects of absenteeism by identifying the short-term costs (below). The short-term costs depend on the time required for the organization to restore itself to normal productivity, which is called the period of friction [29]. It is assumed that productivity losses are limited to the time required to replace the absent workers until the current employees or new workers become productive [30]. Some have criticized that this method underestimates the costs for ignoring the economic costs that other companies paid for losing the employee and recruiting a new employee [27]. Due to that shortcoming, the present study utilizes the lost wages method to estimate the economic costs of absenteeism.

### 2.3. The Cost of Presenteeism

Compared to studies of absenteeism, there have been far fewer studies of presenteeism in both Western and Chinese contexts. Beginning in the 1990s, when many companies restructured, presenteeism was viewed as the consequence of excessive workloads and feelings of job insecurity [31]. Presenteeism refers to the phenomenon of working through illness and injury [32,33], or simply attending work while sick [6]. Cooper [34] argued that sick presenteeism is related to productivity loss because workers who show up sick for work do not perform their tasks efficiently. Thereby, Cooper suggested that presenteeism is even more costly than absenteeism. In the present study, we conceptualize presenteeism in line with Hemp [35], who described it as “being at work, but at the same time not being there”. He advocated the economic advantage of managing presenteeism and estimated that the yearly cost of associated productivity loss is approximately $150 billion in the USA alone. A recent meta-analysis concluded that correlates of working while ill (sick presenteeism) included general ill health, constraints on absenteeism (e.g., strict absence policies and job insecurity), elevated job demands and felt stress, and a lack of job and personal resources (e.g., low support and low optimism) [36]. The authors also reported that absenteeism and sick presenteeism were positively correlated [36]. 

It has been reported that occupational stress, job demands such as time pressure, lack of resources, and unfavorable personal financial circumstances contribute to presenteeism [36,37,38]. These studies have shown a moderate positive correlation between work-related stress and presenteeism that is even greater than absenteeism [39]. Investigations of presenteeism costs related to general health [40] and mental health problems [41] have also shown that these costs are several times higher than the cost of absenteeism. Klein [42] examined the degree of presenteeism and absenteeism among German hospital clinicians, focusing on their correlations with psychosocial stress in the workplace. He found that presenteeism had tighter connections with the psychosocial workload than absenteeism. These workloads involved low rewards, high effort and demands, and increased over-commitment. 

Similar to absenteeism, it is difficult to estimate the prevalence of presenteeism in the workplace or quantify the productivity lost due to it [43,44]. Typically, researchers have examined the costs of presenteeism through self-reported measures or national estimates [12]. The World Health Organization’s Health and Work Performance Questionnaire (HPQ) examines workplace absenteeism, presenteeism, and critical incidents such as accidents and injuries [45]. Although actual financial costs are not directly measured, the prevalence and level of production loss can indirectly contribute to calculating the cost of both absenteeism and presenteeism [12], which is why the HPQ was adopted in this study. 

### 2.4. The Role of Positive Emotions

With the flourishing of positive psychology, researchers have begun searching for factors that can promote people’s well-being and psychological functioning [46]. Positive emotions can buffer the negative economic effects of job stressors. They are defined as temporary psychological, physiological, and facial responses elicited by situations and personal interpretation of the situations [8]. According to Fredrickson’s [8] broaden-and-build theory, positive emotions can widen people’s perception, cognition, and actions and can in turn cultivate their personal resources to better cope with stressors.

In line with broaden-and-build theory, previous research revealed that positive emotions or affects were directly related to better physical health [47,48,49]. In addition, positive emotions were also found to associate with better mental health. Employees who participated in a three-week intervention targeted to improve the positive effect also showed increased levels of self-efficacy, work satisfaction, and mental health [50]. Given that impaired physical and psychological conditions are key reasons for absenteeism and presenteeism [51,52,53,54], positive emotions may lower both by improving people’s health. 

Previous research has supported the beneficial role of positive emotions in organizational outcomes. For instance, the findings of Wright, Cropanzano, and Bonett [55] indicated that positive well-being, similar to positive emotions, could amplify the positive effect of job satisfaction on job performance. Moreover, positive emotions can also alleviate the impact of abusive supervision—a common job stressor—on employees’ turnover intention when coupled with ingratiation behavior [56]. Siu, Cheung, and Lui [57] found that positive emotions were related to higher psychological capital and higher work well-being, thus decreasing employees’ turnover intention. These results are consistent with broaden-and-build theory [8], demonstrating that positive emotions can serve as a psychological resource and build more psychological resources through expanding positive thoughts and beneficial actions.

There is a possibility that positive emotions and job stressors may interact to exert an influence on employees’ absenteeism and presenteeism. Positive emotions were found to foster individuals’ effective coping [58] and proactive behavior [59]. Therefore, employees with higher levels of positive emotions were more likely to view job stressors as challenges and to actively cope with them. In addition, employees with higher positive emotions possessed abundant psychological resources when facing job stressors, which could build their confidence to successfully and flexibly handle them [60]. These two processes may contribute to decreasing employees’ absenteeism and presenteeism. With reference to job demands–resources theory [9], psychological resources may alleviate the detrimental impact of job demands on work performance through decreasing an employee’s strain. Therefore, positive emotions as a psychological resource could be a potential moderator of the negative impact of job stressors on absenteeism or presenteeism. 

### 2.5. The Present Study

**Figure 1:**
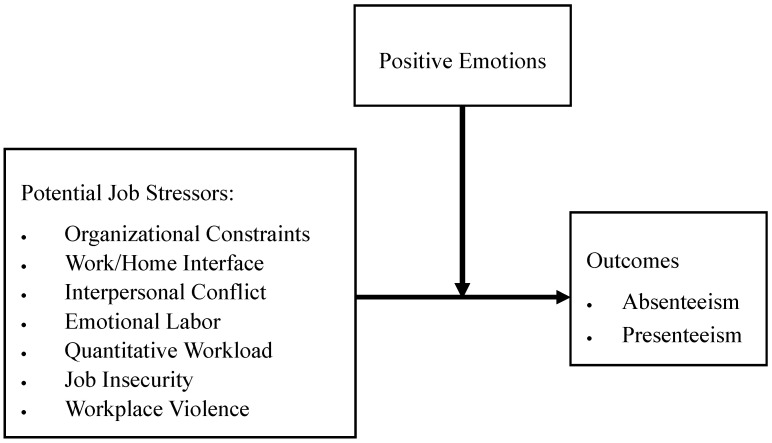
Stressor–outcome model.

The objectives of the current study were, first, to identify and validate major job stressors among Hong Kong employees by using a qualitative method (focus group discussions) and then demonstrate their internal reliability and factor structure with a quantitative method (survey); second, to estimate their economic costs, adding to Western literature; and third, to examine the moderating role that positive emotions play in the job stressor–outcome relationships by a quantitative method (a survey). Previous research in Western and Chinese societies has identified several prominent job stressors, such as quantitative workload, organizational constraints, interpersonal conflicts, emotional labor, job insecurity, the work/home interface, and workplace violence, among others [61,62,63,64,65,66]. In the present study, job stressors were the predictors. In terms of outcome variables, we included absenteeism, presenteeism, and medical expenses [12]. Positive emotions were proposed to be a moderator. Figure 1 presents the stressor–outcome model as the theoretical framework of the survey, with the following hypotheses:

**Hypotheses** **1** **(H1).** *Positive emotions will be negatively related to absenteeism;*

**Hypotheses** **2** **(H2).** *Positive emotions will be negatively related to presenteeism;*

**Hypotheses** **3** **(H3).** *Positive emotions will buffer the negative impact of job stressors on absenteeism, such that the relationship between job stressors and absenteeism will be positive when positive emotions are low, whereas the relationship between job stressors and absenteeism will be negative when positive emotions are high;*

**Hypotheses** **4** **(H4).** *Positive emotions will buffer the negative impact of job stressors on presenteeism, such that the relationship between job stressors and presenteeism will be positive when positive emotions are low, whereas the relationship between job stressors and presenteeism will be negative when positive emotions are high.*

## 3. Method

### 3.1. Sample and Procedure

This study combined qualitative (i.e., focus groups) and quantitative (i.e., survey) methods to examine occupational stress in Hong Kong. Ethical approval for this research was obtained from Lingnan University (EC-038/1718). The purpose of the focus groups was to identify the major job stressors in Hong Kong, which were subsequently examined in a quantitative sample of employees from eight major industries of Hong Kong [67]: Construction; social and personal services (education); social and personal services (health care and social work services); financial and insurance; public administration; accommodation and food services; transportation; and import/export, wholesale, and retail trades.

#### 3.1.1. Focus Group Discussions

To identify the major job stressors in Hong Kong, focus group discussions using critical incident analysis (CIA) [68] with open-ended questions were conducted. A sample item is as follows: “Think of a stressful event that occurred in the last month (if none, what about 3 or 6 months) and describe it in detail, including what led up to it and what happened”. Employees from five companies agreed to participate. They were from four industries, including social and personal services (education), social and personal services (human health and social work services), financial and insurance (two sessions), and information and communications. In each company, six to eight employees, who were either non-managerial staff or front-line supervisors, participated in each focus group. The sessions were tape-recorded. Two staff members from the research team conducted the focus groups. Coupons of HK$100 (US$12.74) were given to the participants as tokens of appreciation for joining the groups. 

#### 3.1.2. The Survey

A purposive sampling method was adopted to recruit the sample, making sure that the participants were drawn from the eight major occupational groups in Hong Kong. Data were collected via the arrangement of a designated person from individual companies and trade unions or associations, including the Hong Kong Federation of Insurers, the Society of Registered Safety Officers, Hong Kong Construction Association, and Hong Kong Construction Industry Employees General Union. Each selected company could choose to adopt an online questionnaire, a printed questionnaire, or both. Some of the companies asked for a stress management seminar to benefit the employees who supported our research. The survey (and the focus group for some companies) was administered before or after the seminar, depending on the company’s request. If the company/organization preferred that we approach the participants directly, we sent our colleagues to the field to collect the data. The participants could choose either a Chinese or English version of the questionnaire. Furthermore, we collected data before stress management seminars targeting employees in the catering industry and construction industry organized by a safety and health organization (the funding body). 

Data were collected from 2082 employees, who returned their questionnaires as either hard or soft copies (powered by Qualtrics software) (see Table 1). After deleting 50 invalid questionnaires (according to the criteria that at least one third was incomplete or contained outliers after data cleaning), the final sample consisted of 2032 participants. 

The ages of the 2032 participants ranged between 18 and 70 (*M* = 37.73, *SD* = 11.13), 54.3% were female (*n* = 1104) and 45.5% were male (*n* = 925), and 51.9% (*n* = 1054) were married/cohabitating and 44.4% (*n* = 902) were single. In terms of the educational level, 52.8% (*n* = 1072) held a university degree or above and 26.5% (*n* = 538) had earned a junior college diploma. Participants’ tenure in their current company ranged from 1 to 480 months (*M* = 6.55 years, *SD* = 7.87 years). In terms of their position, 61.3% of the participants (*n* = 1245) were non-managerial staff, 11.1% (*n* = 226) were frontline supervisors, and 8.6% (*n* = 174) were intermediate managers. In terms of their monthly salary, 25.9% (*n* = 526) of the employees had a current monthly salary in the range of HK$25,000–39,999 and 27.8% (*n* = 565) had a current monthly salary in the range of HK$16,000–24,999. Regarding their lifestyle, 26.1% (*n* = 530) reported that they never did exercise, 33.9% (*n* = 688) of respondents reported that they exercised once a week, 18.7% (*n* = 380) exercised twice a week, 11.3% (*n* = 230) exercised three times a week, 6.2% (*n* = 125) exercised four times a week or more, and 3.5% (*n* = 72) exercised every day.

### 3.2. Measures 

Job stressors. As suggested by the Work Stress Management DIY Kit [63] and previous studies [57,64,65,68], and based on the results obtained from focus group studies (see later section), we included job insecurity, interpersonal conflicts, quantitative workload, organizational constraints, and the work-home interface as job stressors. Unless stated otherwise, a 6-point frequency-based response scale was used for these five job stressors, with 1 = ‘less than once per month’ or ‘never’ and 6 = ‘several times per day’. 

Job insecurity. The three items for this scale were taken from the Work Stress Management DIY Kit of the Occupational Safety and Health Council (OSHC) [63]. Sample items include ‘Your job is insecure’ and ‘Your job is likely to change in the future’. Higher total scores for these three items indicated a higher level of job insecurity. In this study, the internal reliability was 0.66. 

Quantitative workload. The five items for this scale were taken from Spector and Jex’s [69] Quantitative Workload Inventory. Sample items include ‘How often does your job require you to do work very fast?’ and ‘How often does your job require you to work very hard?’. This scale showed a good reliability in previous studies [70]. Likewise, in this study, the internal reliability was 0.92.

Organizational constraints. The 11 items of this scale were taken from Spector and Jex’s [69] Organizational Constraints Scale. The main question was ‘How often do you find it difficult or impossible to do your job because of …?’ Sample answer categories included ‘Poor equipment or supplies’, ‘Organizational rules and procedures’, and ‘Lack of equipment or supplies’. This scale showed a good reliability in previous studies [62,71] and in the current study, the internal reliability was 0.95.

Interpersonal conflicts. Four items were taken from Spector and Jex’s [69] Interpersonal Conflict at Work Scale (ICAWS) for interpersonal conflicts. Sample items include ‘How often do you get into arguments with others at work?’ and ‘How often do other people yell at you at work?’. In this study, the internal reliability was 0.87, consistent with the satisfactory reliability exhibited in previous studies [62,64,71,72].

Work/home interface. The three items for this scale were extracted from the OSHC [63]. Sample items include ‘Your work interferes with home and personal life’ and ‘You are pursuing a career at the expense of home life’. The internal reliability was 0.89, similar to previous studies [73].

Positive emotions. Positive emotions were measured by the 5-item positive emotions scale [57,74]. This is a self-reported assessment that measures employees’ levels of positive emotions, such as happiness, enthusiasm, and excitement. Participants were asked to rate each item using a 6-point Likert scale ranging from “1” = “never” to “6” = “very often”. The scale exhibited a satisfactory internal consistency in previous research [57], as well as in the current research (the internal reliability was 0.91).

Cost indicators. Three cost indicators were used, consisting of absenteeism, presenteeism, and medical expenses per year. We selected the items from the World Health Organization’s Health and Work Performance Questionnaire (HPQ) [45,75] to measure absenteeism and presenteeism. The items for absenteeism were ‘How many work hours does your employer expect you to work in a typical 7-day week?’ and ‘About how many hours altogether did you work in the past four weeks (28 days)?’. The score for absenteeism was calculated by subtracting the exact working hours per month from the expected working hours per month.

As for presenteeism, participants were asked to rate the item ‘How would you rate your overall job performance on the days you worked during the past four weeks (28 days)?’ from “0” to “10”, where 0 is the worst job performance anyone could have at your job and 10 is the performance of a top worker. The score was reversed and transformed into a percentage of presenteeism. For these two measures, we asked the employees to disclose their average salary to transform the absenteeism and presenteeism indicators into costs. The cost of medical treatment was calculated in two parts: The basic cost of seeing a doctor and the cost of sickness due to absence. 

Demographic variables. Demographic information, including age, gender, marital status, education level, tenure in the current company, job position, organizational type, lifestyle (doing exercise), and the industry the employee belonged to, was measured and controlled in the subsequent analyses.

### 3.3. Data Analysis for Focus Group Discussions

The data analysis for focus group discussions was adapted from previous studies [62,76]. After finishing each focus group discussion, the two interviewers listed the most frequent stressful events independently, discussed their results in order to reach an agreement, and produced five focus group reports. Based on these five reports, the first author conducted the content analysis to attribute those stressful events to job stressors, according to the overlap with their definitions [62,64,65,77]. Therefore, several common job stressors for each focus group were identified. By synthesizing the categories of job stressors from the five groups, we could form a better understanding of the prevalent job stressors in Hong Kong. 

### 3.4. Data Analysis for the Survey

The data analysis consisted of correlation analysis, confirmatory factor analysis (CFA), hierarchical regression analysis, a calculation of economic costs, and the latent moderation structural equations method (LMS) [74]. We tested the hypothetical model using SPSS software 23.0 and Mplus 8.0. The results of the correlation analysis provided a preliminary basis for investigating the impact of job stressors on economic outcomes. CFA was adopted to examine the validity and factor structure of the job stressors revealed in focus group discussions, by using the full information maximum likelihood [78]. Subsequently, we conducted hierarchical multiple regression analyses to examine the influences of job stressors on outcomes when controlling for the demographic variables, which allowed us to observe the entire impact of the job stressors. The economic costs of job stressors were then calculated by considering the costs of absenteeism, presenteeism, and medical treatment [12]. LMS was adopted to explore the moderating role of positive emotions in the relationship between job stressors and absenteeism, as well as in the relationship between job stressors and presenteeism. Following Maslowsky et al.’s [79] instructions, item parcels were formed for job stressors and positive emotions. All of the observed variables were standardized before conducting model estimation. The analysis controlled for the effects of the demographic variables, including age, gender, marital status, education level, tenure, lifestyle, position, organizational type, and industry, on absenteeism and presenteeism. Because a model with a latent interaction term (M1) could not be estimated with a normal model parameter output, the model without a latent interaction term (M0) was firstly estimated [79]. If M1 is significantly superior compared to M0, then M1 will fit better than M0.

## 4. Results

### 4.1. Focus Group Discussions

Studying the different industries with their specific work situations allowed us to gain insights into a broad range of job stressors and extract those that concern Hong Kong employees in different sectors. Major findings are summarized in the following.

#### 4.1.1. Social and Personal Services (Education)

Quantitative workload and organizational constraints were prevalent in the education sector, such as enhancing students’ examination achievement, teaching students with special education needs (SEN), preparing examination papers, dealing with parents’ enquiries/requests, and working overtime. Examples of participants’ responses included:

“I am the class teacher who teaches English. I felt stressed about students’ academic results (related to secondary school place allocation).”

“I have a serious SEN student in class. He/she shouts in class during lessons. I have to take care of the discipline and keep the teaching on schedule.”

#### 4.1.2. Social and Personal Services (Health and Social Work Services)

In the health care industry, the major job stressors were quantitative workload (i.e., “long working hours”), organizational constraints (i.e., “lack of manpower”), and interpersonal conflict (i.e., “clients’ rude manners”). Examples of the participants’ responses included:

“I generally (have to work) six continuous days, sometimes up to nine.”

“Clients sometimes kick you or scratch you.”

#### 4.1.3. Financial and Insurance Industry

There were various job stressors in the financial and insurance industry, including job insecurity (i.e., “a change of supervisor”) and quantitative workload (e.g., “time pressure competition among colleagues and communication with colleagues/clients from all around the world when executing global programmes”). Examples of the participants’ responses are as follows:

“The tasks are given very late.”

“I feel stressed when comparing myself with others. Others are doing faster than me.”

#### 4.1.4. Information and Communication Industry

In the information and communication industry, software and techniques have changed rapidly. Employees have to keep learning new programs and techniques to be competitive in the job market. Due to the overtime culture, they felt that they could not leave the office when others were still there. Therefore, those employees were facing stress from quantitative workload, organizational constraints, and the work/home interface. Examples of participants’ responses are:

“The update in software and hardware is too quick. [I] have a feeling of being eliminated/excluded.”

“The supervisor gave complicated tasks, but the time is insufficient.”

Overall, based on the findings obtained from the focus group discussions in different industries, the most common job stressors included job insecurity, organizational constraints, interpersonal conflict, quantitative workload, and the work/home interface, corroborated by previous studies of Chinese societies [63,64,65]. However, unlike Western societies, emotional labor and workplace violence were not found to be frequent stressors [1,61].

### 4.2. The Survey

#### 4.2.1. Testing Normal Distributions of Variables

The skewness of each study variable ranged from −0.59 to 1.34 (job insecurity was 1.34 and interpersonal conflict was 1.11; however, the attributes of these two variables in our model were predictors. The PP Plot of the standardized residuals of the separate outcomes seemed fine, so the distribution of residuals of the outcomes followed a normal distribution). Kurtosis displayed values between −0.65 and 1.40, indicating that the study variables fulfilled the normal distribution assumptions. The tolerance was between 0.34 and 0.95, which was greater than the lower threshold of 0.2 [80]. Furthermore, the variance inflation factor (VIF) was between 1.09 and 2.96, and all of the values were lower than the upper threshold of 10 [81].

#### 4.2.2. Factor Structure of Job Stressors

The result of CFA with five job stressors exhibited a satisfactory model fit: *χ*^2^(289) = 2724.664; RMSEA = 0.064; 90% CI = [0.062, 0.067]; SRMR = 0.042; CFI = 0.930; and TLI = 0.921. Specifically, for the five job stressors, all of the factor loadings were significant, *p* < 0.001. Nearly all the standardized factor loadings of items for each job stressor were larger than 0.600, except for item 3 for job insecurity (factor loading = 0.465, *p* < 0.001). Therefore, the result of CFA supported the validity evidence of the results of focus group discussions. 

Table 2 presents the means, standard deviations, and correlations among the research variables. The results showed that job insecurity was positively associated with absenteeism (*r* = 0.08, *p* < 0.001). The work/home interface (*r* = 0.14, *p* < 0.001), job insecurity (*r* = 0.22, *p* < 0.001), interpersonal conflict (*r* = 0.17, *p* < 0.001), quantitative workload (*r* = 0.09, *p* < 0.001), and organizational constraints (*r* = 0.18, *p* < 0.001) were significantly related to presenteeism. The correlation between positive emotions and absenteeism was non-significant, *p* > 0.05. Therefore, the result fails to support H1. In addition, positive emotions were negatively correlated with presenteeism (*r* = −0.23, *p* < 0.001). Therefore, H2 is supported. Table 2 also shows that absenteeism and presenteeism were not significantly correlated. 

The results of hierarchical regression analyses for the impact of the five job stressors on absenteeism and presenteeism are depicted in Table 3. Specifically, after controlling for the influences of the nine demographic variables, five job stressors significantly predicted absenteeism (Δ*R^2^* = 0.03, *p* < 0.001) and presenteeism (Δ*R^2^* = 0.05, *p* < 0.001). Specifically, three of the stressors (the work/home interface, job insecurity, and quantitative workload) significantly predicted absenteeism. Four of the stressors (job insecurity, interpersonal conflict, quantitative workload, and organizational constraints) significantly predicted presenteeism. The results showed that the stressors generally had a significant effect on economic outcomes when controlling for the influence of the demographic variables.

To quantify the economic costs to society from occupational stress, two main economic outcomes were considered: Absenteeism and presenteeism. Additionally, medical treatments (leading to medical expenses) due to job stressors were taken into account. The detailed calculations are presented in the following.

### 4.3. Calculating the Cost of Absenteeism

The costing tool was retrieved from the National Institute for Health and Clinical Excellence [70] Public Health Guidance 22. We amended the context of the calculation elements to fit the circumstances of the Hong Kong workplace.

For absenteeism:

(a) Calculating the average number of absences in days per month (from the WHO scale), equal to M (average number of absenteeism hours in four weeks; the value is 4.72, which is captured in Table 4)/M (work hours that your employer expects you to work in a typical week/7; the value captured from the WHO scale is 48/7), namely, 4.72/(48/7) = 0.69 day;
(b) Estimating the variance of absenteeism attributable to the five job stressors. The value was captured in the hierarchical regression results depicted in Table 3. In this study, the figure was 3%;
(c) The cost attributable to absenteeism each day could be roughly estimated by the employees’ average daily salary. In this study, the median monthly salary was HK$16,000 to HK$24,999 (4 weeks) or HK$571.43 to HK$892.82 in terms of the daily salary.

Finally, the annual cost of absenteeism attributable to work stressors equaled (a) × (b) × (c) × 12. In our sample, the figure would be $HK141.94 to HK$221.78, representing the annual cost per employee due to absenteeism. Projecting this to the entire employee population (3.87 million) [82] of Hong Kong, the annual cost can be estimated to be $HK549 million (USD 70.2 million) to HK$858 million (USD 109.8 million) (see Table 4).

Using the median monthly wage of working people in Hong Kong [67], which was HK$17,500 (4 weeks), or $HK625 in terms of the daily salary, the annual cost due to absenteeism among the entire employee population would be HK$600.82 million.

We performed a check of the absenteeism data. In our questionnaire, we asked about absenteeism in a week and four weeks. We checked to see whether there was any discrepancy between the participants’ answers for these two questions. After checking, we suspected fake data and deleted them accordingly. In the end, 1253 questionnaires were deemed to be credible enough to estimate absenteeism. 

### 4.4. Calculating the Cost of Presenteeism

The rationale for calculating the cost of presenteeism was similar to calculating the cost of absenteeism:

(a) Calculating the average number of days of presenteeism per month. Because such data are difficult to measure, we adopted the recommendation of the Sainsbury Centre for Mental Health. The UK report showed that the estimated ratio of presenteeism to absenteeism was 1.5. [83], p. 1. The number in our study was 1.035 days (0.69 × 1.5);
(b) Estimating the variance of presenteeism attributable to the five job stressors. The value was captured through hierarchical regression, as seen in Table 3. In this study, the figure was 5%;
(c) The cost attributable to presenteeism each day could be roughly estimated by the employees’ average daily salary. In this study, the median monthly salary was HK$16,000 to HK$24,999 (4 weeks) or HK$571.43 to HK$892.82 in terms of the daily salary.

Finally, the annual cost of presenteeism attributable to job stressors equaled (a) × (b) × (c) × 12. In our sample, the figure was HK$354.86 to HK$554.44, representing the annual cost per employee due to presenteeism. Projecting this to the entire employee population of Hong Kong (3.87 million) [84], the annual cost would be HK$1373.30 million (USD 175.3 million) to HK$2145.69 million (USD 274 million) (Table 4).

If we use the median monthly wage of working people in Hong Kong, [67], which was HK$17,500 (4 weeks), or HK$625 in terms of the daily salary, the annual cost due to presenteeism for the entire employee population of Hong Kong would be HK$1.50 billion.

### 4.5. Calculating the Cost of Medical Treatment (Medical Expenses)

Based on the actual situation, the cost of medical treatment was calculated in two parts: The basic cost of seeing a doctor and the cost of absence due to sickness.

For the basic cost of seeing a doctor:

(a) Calculating the average number of times an employee sees a doctor in one year (the value was captured from the WHO scale). In our sample, the number was six.
(b) Estimating the basic cost of treatment at public clinics. The government provides public healthcare to all residents of Hong Kong, including non-permanent residents. Hong Kong has a comprehensive public healthcare system, including hospitals, clinics, and specialist care. The fees for visiting an Accident & Emergency (A & E) department under the Hospital Authority (HA) are regulated by the government and are very moderate, especially when compared to the prices of private clinics. Effective from 18 June 2017, in general, eligible persons (e.g., people with a Hong Kong ID) pay HK$180 for one visit to an A & E. Therefore, we adopted HK$180 as the basic cost for one visit to a public clinic (note that the cost for private clinics is much higher). Because the government may sponsor the HA’s expenses, to capture more realistic expenses, we also considered the fees for a non-eligible person, which are HK$1230 per visit [85].

The basic cost of seeing a doctor equaled (a) × (b). In our sample, the figure would be HK$1080 to HK$7380, representing the annual cost per employee to see a doctor.

For the cost of sickness absence:

(c) The cost attributable to sickness absence each day could be roughly estimated by the employees’ average daily salary. In this study, the median monthly salary was HK$16,000 to HK$24,999 (4 weeks) or HK$571.43 to HK$892.82 in terms of the daily salary;
(d) Calculating the average number of sickness absence days in one year. In our sample, the number was 3.2. Please note that the 1995 data were credible enough to estimate the sickness absence after checking;
(e) The cost of sickness absence equaled (c) × (d). In our sample, the figure would be HK$1828.58 to HK$2,857.02, which represents the annual cost per employee due to sickness absence.

Hoel, Sparks, and Cooper [86] estimated that 30% of sickness absence is directly caused by stress. Finally, the annual cost of medical treatment attributable to stress per employee equaled {(a) × (b) + (c) × (d)} × 30%. In our sample, the figure would be HK$872.57 to HK$3071.11, which represents the annual cost per employee due to medical treatments. Projecting this to the entire employee population in Hong Kong (3.87 million) [84], the annual cost would be HK$3376.85 million (USD 368.9 million) to HK$11,885.19 million (USD 521.2 million) (see Table 4).

If we use the median monthly wage of working people in Hong Kong [67], which was HK$17,500 (4 weeks), or HK$625 in terms of the daily salary, the annual cost due to medical treatments for the entire employee population would be HK$3.58billion to HK$10.89 billion.

#### Total Economic Cost of Stress

The annual total economic cost of stress is equal to the sum of the above three terms (absenteeism, presenteeism, and medical expenses), which is approximately HK$5.30 billion (USD 614 million) to HK$14.89 billion (USD 905.3 million) (see Table 4). However, the actual costs are much higher than this estimate, for two reasons.

First, many factors, such as turnover, premature retirement, and damage to equipment and production resulting from too much stress, are not included [86]. These factors, as mentioned above, are huge economic costs to society when they are added together. Second, we only used the base values of each indicator (medical expense) when we assessed the cost of occupational stress. The real value could be much higher. According to the most updated figures from the Census and Statistics Department [87], Hong Kong’s GDP (constant LCU) in 2018 was HK$2845 billion. The estimated cost of stress was about 0.19%–0.52% of GDP.

**Figure 2:**
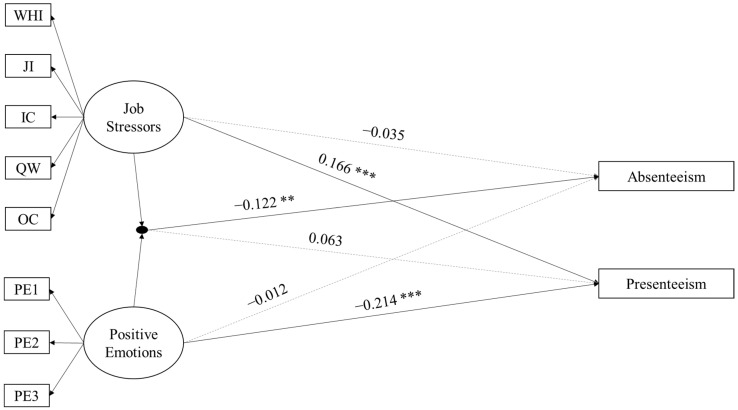
The moderating role of positive emotions in the relationships between job stressors and absenteeism/presenteeism. *Note.* * *p* < 0.05; ** *p* < 0.01; *** *p* < 0.001. WHI = work/home interface; JI = job insecurity; IC = interpersonal conflict; QW = quantitative workload; OC = organizational constraints.

For the direct and moderating effects of positive emotions in the relationships between job stressors and absenteeism or presenteeism, the model with the main effects of job stressors and positive emotions on absenteeism and presenteeism (M0) fitted well (*χ^2^* = 704.742, *df* = 103, *p* < 0.001, CFI = 0.931, TLI = 0.910, SRMR = 0.055, RMSEA = 0.057, 90% CI = [0.053, 0.061], *p* = 0.002). Next, the model including the latent interaction term (M1) showed significant model fit compared with M0 (*D* (2) = 11.192, *p* < 0.005). Therefore, M1 was chosen in this step. The structural model of M1 is shown in Figure 2. Specifically, the effect of the interaction term of job stressors and positive emotions on absenteeism was significant (*b* = −0.122, *se* = 0.046, *p* = 0.008). However, the effect of the interaction term of job stressors and positive emotions on presenteeism was non-significant (*b* = 0.063, *se* = 0.033, *p* > 0.05). For the moderating effect of positive emotions in the relationship between job stressors and absenteeism, when positive emotions were at the -2SD level, job stressors significantly and positively associated with absenteeism (*b* = 0.178, *se* = 0.086, *p* = 0.039); when positive emotions were at the -1SD level, the relationship between job stressors and absenteeism was non-significant (*b* = 0.071, *se* = 0.057, *p* = 0.214); when positive emotions were at a medium level, the relationship between job stressors and absenteeism was non-significant (*b* = −0.035, *se* = 0.049, *p* = 0.482); when positive emotions were at the +1SD level, job stressors negatively associated with absenteeism (*b* = −0.141, *se* = 0.069, *p* = 0.042); and when positive emotions were at the +2SD level, job stressors negatively associated with absenteeism (*b* = −0.247, *se* = 0.102, *p* = 0.015), suggesting a buffering effect. For the effects of job stressors and positive emotions on presenteeism, job stressors were significantly and positively related to presenteeism (*b* = 0.166, *se* = 0.036, *p* < 0.001). Positive emotions significantly and negatively associated with presenteeism (*b* = −0.214, *se* = 0.030, *p* < 0.001). Therefore, H3 is supported and H4 is not supported by the results.

## 5. Discussion

Through the qualitative and quantitative approaches adopted in this study, we identified and validated five major job stressors perceived by Hong Kong employees (quantitative workload, job insecurity, interpersonal conflict, the work/home interface, and organizational constraints), their economic cost, and the beneficial role of positive emotions in alleviating the economic costs of job stressors. In line with the literature on Western societies, the negative impact of occupational stress on employees could lead to huge economic effects. The annual total economic cost of work stress equal to the sum of absenteeism, presenteeism, and medical treatment is approximately HK$4.81 billion to HK$7.09 billion (USD 614 million to USD 905.3 million). This cost is about 0.19–0.52% of Hong Kong’s GDP, which is lower than that of some Western countries, such as the UK [83], the USA [10], and Germany [88]. However, it is important to point out that the economic costs of stress are based on different indicators, depending on the country. For example, in France, the total cost of occupational stress is estimated based on costs for healthcare, absenteeism, a loss of activity, and a loss of productivity due to premature death [84]. In Germany, the estimated cost of occupational stress in 2008 is based on direct costs, such as prevention, rehabilitation, maintenance treatment, and administration, as well as indirect costs, such as lost working years through incapacity, disability, and premature death [88].

It should be noted that absence from work might involve both absenteeism and medical costs. However, their implications are quite different. In this study, we also took special considerations in the measurement and calculation processes. We assumed that the cost of absenteeism would be independent of medical costs for three reasons. First, the amount of time of absenteeism is a generalized concept. Absolute absence (‘working hours that the employer expected you to work a week’ minus ‘working hours that you have worked a week’) was used here. The amount of time of absenteeism was based on an overall evaluation, including vacation, loafing on the job, mind wandering, late arrival, early departure, and so on. In contrast, the absence time associated with medical cost represents a narrow use of the concept and is limited to sick leave. Like the tip of an iceberg, sick leave is the most evident, but smallest, part of absence. The estimates of variance based on empirical data are also consistent with our assumption. Three percent of absolute absence was attributable to job stressors in this study, whereas, according to Hoel et al. [86], 30% of sickness absence is directly caused by stress. Therefore, compared to the effects of stress on medical costs, the effects of absenteeism are relatively limited, and the costs would be substantially reduced. 

Second, the items of measurement were deliberately designed for different scales. That is, a week was used for absenteeism and three months was used for medical costs. Third, the two costs were calculated by different items. The items for absenteeism were ‘working hours that the employer expected you to work a week’ and ‘working hours that you have worked a week’. The item for medical treatment was ‘the average number of days of sick leave in the last three months’. To sum up, given the great difference between absolute absence and sickness absence, with the follow-up calculation processing, the overlap could have been inadvertent and ignored. Furthermore, one could argue that there might be overlap for absenteeism and presenteeism estimates because there is a positive correlation between absenteeism and sick presenteeism [36]. However, we found no significant correlation between absenteeism and presenteeism in this study because we did not conceptualize presenteeism as ‘working while sick’. Future research should confirm these results by adding sick presenteeism and estimate the degree to which the cost of absenteeism can overlap with medical costs and presenteeism. Concerning job stressors, unlike Western societies, emotional labor and workplace violence were not found to be frequent stressors [1,61]. This might be due to the fact that only five focus groups from four industries were included. Future research should conduct more interviews to generate more qualitative data.

As for the beneficial role of positive emotions, the present research found that positive emotions could alleviate the economic costs of absenteeism associated with job stressors. According to broaden-and-build theory [8], positive emotions accumulate an individual’s psychological resources, which enable them to treat job stressors as challenges so as to build subsequent psychological resources instead of being threatening and losing psychological resources. Siu et al. [57] found that higher positive emotions were associated with a higher level of psychological capital. Therefore, there is a possibility that positive emotions may buffer the negative impact of job stressors by cultivating employees’ psychological capital. Future research could explore the potential role that psychological capital plays or other underlying mechanisms in the buffering effect of positive emotions in the relationship between job stressors and absenteeism.

The current research also found that positive emotions directly decreased the economic costs of presenteeism. This finding was in accordance with previous findings that positive emotions decreased negative organizational outcomes such as turnover intention [57]. Employees with a high level of positive emotions were less likely to work in a state of presenteeism more positive emotions may directly lead to better well-being [49,50] and mean that employees are more likely to engage in work with great vigor, dedication, and absorption [89]. Future research could further explore the mechanism of the direct and indirect effects of positive emotions on presenteeism.

### 5.1. Methodological and Theoretical Contributions of the Study

This is one of very few studies to have adopted both qualitative and quantitative approaches when examining the economic cost of occupational stress and provides validation evidence of five important job stressors (as antecedents) relevant to both Western and Chinese societies, adding to its methodological contribution. Findings from the current study have filled a gap in the knowledge by estimating the economic cost of occupational stress (in monetary terms) in a Chinese society, representing a less explored region, as reviewed by Hassard et al. [1]. Furthermore, this study contributes to theories in occupational stress by studying stress-related absenteeism and presenteeism simultaneously to estimate the economic cost, adding to recent literature reviews [1] and a meta-analysis [36]. We have also demonstrated that positive emotions, representing a less explored individual factor, are a significant moderator in alleviating the impact of stress on economic outcomes, adding to the theoretical contribution to occupational health psychology. 

### 5.2. Practical Implications for Occupational Health Psychology

The five validated job stressor scales can be used for academic research in occupational health psychology and intervention work in the workplace. Managers and employees should both diagnose the different kinds of job stressors so that they can make accurate judgements of the prevalent occupational stress situations. The huge cost of occupational stress estimated by the study could serve as an alarm to owners and HR practitioners in Hong Kong and other Asian societies. To reduce the economic costs of an organization caused by occupational stress, organizational managers and HR managers should pay special attention to the stressors of job insecurity, the work/home interface, interpersonal conflict, quantitative workload, and organizational constraints. They should take measures, such as enhancing the work/life balance, updating equipment or supplies, providing sufficient training and guidance, and providing adequate support, to reduce employees’ job insecurity to a reasonable level and minimize the effects induced by organizational constraints. Many organizations and employees could underestimate the impact of occupational stress. Organizations and individuals should comprehensively review the impact of occupational stress induced by the workload and home/work interface. They should pay attention to the impact of job stressors and begin intervention work on effective stress management. This, in turn, would help companies/organizations to effectively tackle organizational problems and eventually reduce economic costs.

Furthermore, exploring the buffering effect, as well as the main effect, of positive emotions on absenteeism and presenteeism provides practical implications for highlighting the importance of positive emotions in organizational settings. Previous research provides suggestions on how positive emotions as a psychological resource can be fostered. For instance, Fredrickson [90] has listed several interventions, such as meditation, relaxation exercises, imagery exercises, and exercises aimed at finding positive meaning, that could cultivate an individual’s positive emotions. Loving-kindness meditation was also found to increase positive emotions [91]. Our research suggested that interventions targeted at promoting individuals’ positive emotions may be effective in terms of reducing the economic costs elicited by job stressors. This provides preliminary evidence to encourage employers to utilize these interventions so as to produce positive change in workplaces which, in turn, enhance occupational health.

### 5.3. Limitations of the Study

The measurement approach used by the study to calculate absenteeism can be improved by defining absolute absenteeism, relative absenteeism, and relative hours of work more clearly. The conceptualization and measure of presenteeism were different from sick presenteeism, which has been more widely used in recent studies. One may argue that the WHO’s HPQ used in the present study could be interpreted as a performance measure. Future studies should adopt a more well-defined concept and measure of presenteeism, and more work can be done on sickness absence culture [92]. 

Another limitation of the current study is the adoption of a purposive sampling method. Even though we showed the sample in the survey more or less representing the major industries in Hong Kong and conducted analyses illustrating that the study variables basically could fulfil the normal distribution assumptions, it is possible that this sampling approach could limit the generalizability of our estimates to the societal level. Furthermore, even though the sample size employed for the validation of job stressor scales is adequate, only about 50% of the collected data could be used to estimate absenteeism. An interpretation of the reliability and validity of the results should be done with caution. Lastly, we mainly followed the three important measures of economic costs of occupational stress, as summarized by the European Agency for Safety and Health at Work [12]. Referring to a recent discussion on the conceptual framework of estimating the total cost of stress [1], we only estimated two indirect costs and one direct cost. Future research should examine what is missing in the estimates, such as aggression and violence in the workplace [61]. Moreover, the results of this research call for the design and validation of workplace interventions in Hong Kong and other Asian societies. 

## 6. Conclusions

This research has demonstrated the enormous monetary costs associated with workplace stress, which further carry tremendous health-impairment burdens for individual employees [2]. With this study, we further highlighted the five major stressors for Hong Kong employees: Job insecurity; quantitative workload; organizational constraints; interpersonal conflicts; and the work/home interface. Targeting these workplace stressors will likely result in a direct cost reduction for Hong Kong employers. Furthermore, the findings of this study show that positive emotions can be leveraged to guard against the negative outcomes of workplace stress. 

## Figures and Tables

**Table 1:** Sample distributions in eight major industries in Hong Kong.

Variable.	Category	N	Percentage	No. and Percentage of Persons Engaged (Dec. 2017) (Except Public Administration) ^1^
Industries	Construction	205	10.1	122,761 ^2^ (4.3%)
	Education	331	16.3	201,488 (7.1%)
	Human health and social work service	280	13.8	158,606 (5.5%)
	Financing and insurance	336	16.5	227,156 (7.9%)
	Public administration (including government administration, economic and social policy, public order, and safety activities)	150	7.4	111,009 (2.9%) ^3^
	Accommodation and food services	124	6.1	283,505 (9.9%)
	Transportation	200	9.8	85,191 (3.0%)
	Import/export, wholesale, and retail trades	149	7.3	807,499 (28.3%)
	Information and communications	18	0.9	107,122 (3.7%)
	Real estate	46	2.3	131,855 (4.6%)
	Social and personal services (other services)	37	1.8	76,866 (2.7%)
	Professional and business services (Professional, scientific, and technical services and administrative and support services)	44	2.2	377,659 (13.2%)
	Others (like manufacturing, sports, supply, arts, NGO, etc.)	40	2.0	
	Missing	72	3.5	

*Note.*^1^. The material retrieved from the Quarterly Report of Employment and Vacancies Statistics in December 2017 reported by the Census and Statistics Department, Hong Kong Special Administrative Region; ^2^. Construction sites include manual workers only; ^3^. The material retrieved from Hong Kong for figures from 2018 reported by the Census and Statistics Department, Hong Kong Special Administrative Region.

**Table 2:** Means, standard deviations, and correlations among the main variables.

.	*M*	*SD*	1	2	3	4	5	6	7	8
1. Work/Home Interface	2.53	1.25	1							
2. Job Insecurity	1.89	1.00	0.48 ***	1						
3. Interpersonal Conflict	2.13	1.09	0.53 ***	0.44 ***	1					
4. Quantitative Workload	2.68	1.25	0.66 ***	0.44 ***	0.54 ***	1				
5. Organizational Constraints	2.44	1.13	0.59 ***	0.49 ***	0.67 ***	0.66 ***	1			
6. Positive Emotions	3.50	0.97	−0.29 ***	−0.18 ***	−0.25 ***	−0.24 ***	−0.33 ***	1		
7. Absenteeism	4.72	46.74	−0.02	0.08 ***	0.03	−0.01	0.04	−0.04	1	
8. Presenteeism	0.30	0.15	0.14 ***	0.22 ***	0.17 ***	0.09 ***	0.18 ***	-0.23 ***	0.04	1

*Note*. * *p* < 0.05; ** *p* < 0.01; *** *p* < 0.001.

**Table 3:** Hierarchical multiple regression results of job stressors on economic outcomes.

Predictor.	Absenteeism		Presenteeism	
	Model 1	Model 2	Model 1	Model 2
Age	−0.02	−0.03	−0.09 **	−0.09 **
Gender	0.05	0.05	−0.01	0.01
Marital Status	0.04	0.05	0.03	0.02
Education Level	0.00	−0.01	−0.05 *	−0.04
Tenure	0.06	0.08 *	−0.10 ***	−0.07 *
Position	−0.03	0.00	−0.06 *	−0.04
Organization Type	−0.09 **	−0.07 *	−0.02	−0.01
Lifestyle	−0.01	−0.02	−0.05 *	−0.03
Industry	0.03	0.03	0.00	0.00
Δ*R^2^*	0.02 *		0.04 ***	
Work/Home Interface		−0.13 **		0.01
Job Insecurity		0.13 ***		0.16 ***
Interpersonal Conflict		0.02		0.06 *
Quantitative Workload		−0.10 *		−0.08 *
Organizational Constraints		0.08		0.09 *
Δ*R^2^*		0.03 ***		0.05 ***

*Note*. Values are standardized beta coefficients. * *p* < 0.05; ** *p* < 0.01; *** *p* < 0.001.

**Table 4:** Summary of costs.

Source.	Formula	a	b	c	d	HK Population of Employees (Million)	Total (Million HKD)
Absenteeism	(a) × (b) × (c) × 12 × HK population	0.69	3%	571.43–892.82	N/A	3.87	549.32–858.28
	census data			625			600.82
Presenteeism	(a) × (b) × (c) × 12 × HK population	1.035	5%	571.43–892.82	N/A	3.87	1373.30–2145.69
	census data			625			1502.04
Medial Cost	{(a) × (b) + (c) × (d)} × 30% × HK population	6	180–1230	571.43–892.82	3.2	3.87	3376.85−11,885.19
	census data			625			3575.88−10,890.18
Total	Based on sample data						5299.47–14,889.16
	Based on census data						5678.74–12,993.04

*Note.* For details on (a), (b), (c), and (d), please refer to explanations in the text.
